# Effect of Long-Term Moderate Physical Exercise on Irisin between Normal Weight and Obese Men

**DOI:** 10.1155/2020/1897027

**Published:** 2020-09-01

**Authors:** Farah A. Rashid, Hamid Jaddoa Abbas, Naser Ali Naser, Hana'a Addai Ali

**Affiliations:** ^1^Department of Chemistry, College of Science, Al-Nahrain University, Baghdad, Iraq; ^2^Alzahraa Medical College, Basrah, Iraq; ^3^Al-Faiha'a Teaching Hospital, Basrah, Iraq; ^4^Department of Chemistry, College of Science, University of Kufa, Najaf, Iraq

## Abstract

**Background:**

Irisin is a myokine that has a beneficial effect on obesity and glucose metabolism by increasing energy expenditure. This study aims to investigate the effect of long-term moderate physical exercise on irisin levels and its correlations with body mass index (BMI), waist circumferences (WC), and metabolic parameters in normal weight and obese males. *Material and method*. A follow-up case-control study of sixty male participants, comprised of thirty normal weight and thirty obese, who had undergone supervised long-term moderate physical exercises for six months. Serum irisin levels, fasting blood glucose, serum insulin, homeostatic model assessment of the insulin resistance index (HOMA-IR), and *β*-cell function (HOMA-B_2_) were assessed.

**Results:**

Long-term moderate exercise induced elevation of the irisin level significantly (*P* < 0.0001) with significant reduction of the BMI, WC, fasting blood glucose, insulin, HOMA-IR, and HOMA-B_2_ levels (*P* < 0.0001) in comparison between obese and normal weight groups. There are significant differences for each parameter in each obese and normal weight group before and after physical exercise with exception of the BMI and WC in the normal group. Significant negative correlations were shown between irisin and blood glucose and insulin and HOMA-IR levels in the obese group and normal weight group.

**Conclusion:**

Irisin improves glucose homeostasis after long-term moderate physical exercises, suggesting that irisin could have regulatory effect on glucose, insulin resistance, and obesity and it could be used as a potential therapy for obesity and insulin resistance.

## 1. Introduction

Obesity is one of the most interesting epidemic issues worldwide. It is considered one of the most important risk factors for several diseases including cardiovascular diseases, type 2 diabetes mellitus, respiratory disorders, and some types of cancer [1, 2]. Adipose tissue is considered as an endocrine organ that regulates energy homeostasis and inflammation [3]. There are two types of adipocytes: fat-storing white adipocytes and thermogenic brown adipocytes. In adult humans, a white adipocyte converts to brown adipocyte in a process called browning. The brown adipocytes are active energy consumers and abundant in the mitochondria. In contrast, the white adipocytes conserve fatty acids and triglycerides with a very few mitochondria and secrete many adipokines [4, 5]. It is thought that brown adipose tissue is a promising target therapy for metabolic diseases and obesity. Physical exercises stimulate browning of white adipose tissue. There are many myokine, adipokine, and hepatokine factors that can be induced during the browning process [6].

One of the recent discovered myokines is irisin. Irisin has an interest of many researchers due to its important role in obesity. Irisin hormone is a small peptide that originates from the proteolytic cleavage of the fibronectin type III domain containing protein 5 (FNDC5). FNDC5 is a type I trans-membrane protein which is predominantly present in cardiac and skeletal muscles and is secreted into the blood circulation [7]. Recently, a meta-analysis study found that the irisin level is higher in obese than normal weight individuals [8]. It is believed that irisin has a significant role in exercise-induced browning of white adipose tissues and stimulating the metabolic gene expression. In the cell line model, it was found that treatment of myocytes C2C12 with irisin led to increased oxidative metabolism and mitochondria biogenesis [9]. The role of irisin is ambiguous, since it differs with different experimental conditions. Some studies reported that circulating irisin is not changed by exercises [10, 11]. On the other hand, Bostrom found that the irisin level increased with exercise for 12 and 10 weeks in humans and mice, respectively [7]. Huh et al. showed that circulating irisin is increased by acute exercise for 8 weeks in nonobese healthy male [12]. However, no pervious study investigates the effect of the irisin level through long-term moderate physical exercise in obese and healthy individuals and whether or not exercise-induced irisin could improve insulin/glucose homeostasis. Thus, the current study aims are to investigate the effect of six months of regular moderate exercises on irisin levels in obese and normal weight male and study whether the elevation in the irisin level could be correlated with metabolic variables.

## 2. Materials and Methods

### 2.1. Study Design

A follow-up case-control study was conducted within Al-Sadder medical City, Al-Najef, Iraq, during the period from December 2018–June 2019. Male participants, aged from 20–43 years, were randomly and newly selected from the attendance of long-term moderate training programs. All study participants were informed about the physical training protocol and gave written consent for their participation. The protocol of the study was approved by the Kufa Medical College Ethical Committee. The participants were comprised of two groups: a normal weight group (30 participants with BMI <25 kg/m^2^) and an obese group (30 participants with BMI ≥30 kg/m^2^). Obese participants who follow a weight-loss diet regimen during the study were excluded. The participants with current and chronic medical illnesses were excluded. All participants attended a supervised training program, one session per day for six months. Every moderate session was taken for 3 hours. The training program was started with a warm-up period performed at a low load. Then, an active phase was gradually increased by increasing the heaviness of loading gradually, and the program was ended with a recovery and relaxation phase [13]. For all participants, body weight was measured before and after the exercise program with highly precise electronic scales (Seca, Germany). Height was measured in a standing position without shoes by using a fixed stadiometer (Seca, Germany). Waist circumferences (WC) were measured as described in [14]. The evaluation of the physical training program involved four assessments, and each one was conducted after two months. The first, second, third, and fourth assessments were conducted at zero, second, fourth, and sixth months of the physical training program, respectively.

### 2.2. Sample Collection

Five ml of fasting blood samples was collected by using a standard venipuncture at the zero, second, fourth, and sixth months of the study. The blood was centrifuged at 2,200*g* for 10 minutes and stored at −80°C until biochemical analysis. Serum irisin levels were analyzed by using an enzyme-linked immunosorbent assay (ELISA) kit (Phoenix Pharmaceuticals Inc., CA, USA). Insulin levels were measured by the chemiluminescence technique (Roche, Cobas E 411). Glucose levels were measured by the glucose enzymatic method (Roche, Cobas Integra 400). The homeostasis model assessment of insulin resistance (HOMA-IR) was calculated depending on the formula: HOMA-IR = fasting serum glucose (mg/dl) × fasting serum insulin (*μ*IU/ml)/405. The homeostasis model assessment of *β*-cell function (HOMA-B) was calculated using (20 × fasting serum insulin)/(FPG −3.5) and used to represent *β*-cell function [15].

### 2.3. Statistics Analysis

Statistical analysis of the study was performed using SPSS for Windows, Version 20.0 (SPSS Inc., Chicago, IL, USA). The continuous variables were presented as mean ± SD. Unpaired Student's *t*-test was preformed to evaluate the significant differences between the normal weight and obese group in each two months and before and after six months of physical exercise. Linear regression was preformed to test the increasing and decreasing of each continuous variable during six months of physical exercise. The correlation between exercise-induced irisin and metabolic parameters was assessed by Pearson's correlation. The statistical significance was set at a *P* value less than or equal to 0.05.

## 3. Results

### 3.1. Comparison between Metabolic Parameters in Normal Weight and Obese during Six Months

Metabolic parameters, BMI, WC, FBS, insulin, HOMA-IR, HOMA-B_2_, and irisin levels, were compared between normal weight and obese in 0, 2, 4, and 6 months of moderate physical exercise (Table 1). There was no significant difference in the mean of ages between normal weight and obese groups, 34.10 ± 5.51 and 34.33 ± 4.85, *P*=0.376, respectively. However, significant differences were found between the normal weight group and obese group in the BMI, WC, FBS, insulin levels, HOMA-IR, HOMA-B_2_, and irisin. Interestingly, there were significant decreases in each of insulin, HOMA-IR, and HOMA-B_2_ and a significant increase in the serum irisin concentration with increased duration of exercise in both groups.

### 3.2. Comparison in the Level of Irisin and Metabolic Variables before and after Physical Exercises

To test the effect of six months of physical exercises on each irisin and metabolic variables, the comparison between before and after moderate physical exercises of parameters were tested in the normal weight and obese group. As it is shown in Table 2, a highly significant increase was found in the irisin concentration when compared between the irisin level before and after physical exercises for six months for each normal weight and obese groups. In contrast, a highly significant decrease was found in FBS, insulin, an HOMA-IR in comparison between before and after physical exercise for each normal weight and obese groups. Although there were no significant differences in the BMI and WC in the normal weight group after 6 months of exercises, a high significant difference was found in the obese group. Interestingly, there is a significant increase in HOMA-B_2_ in the normal group and a significant decrease in HOMA-B_2_ in the obese group.

### 3.3. Relationship between Irisin and Metabolic Variables

**Figure 1:**
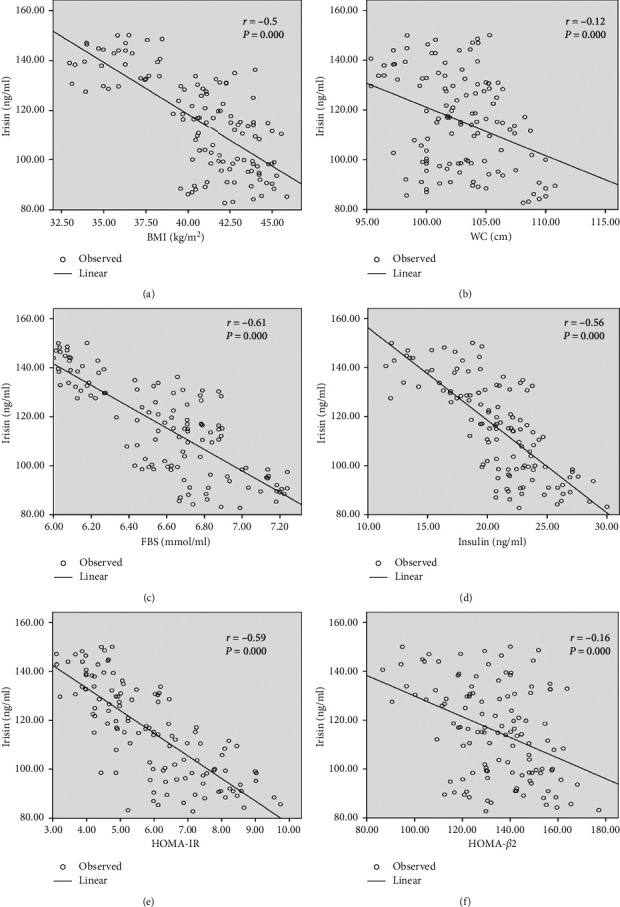
Correlation between irisin and metabolic variables after six months of moderate physical exercise in the obese group.

**Figure 2:**
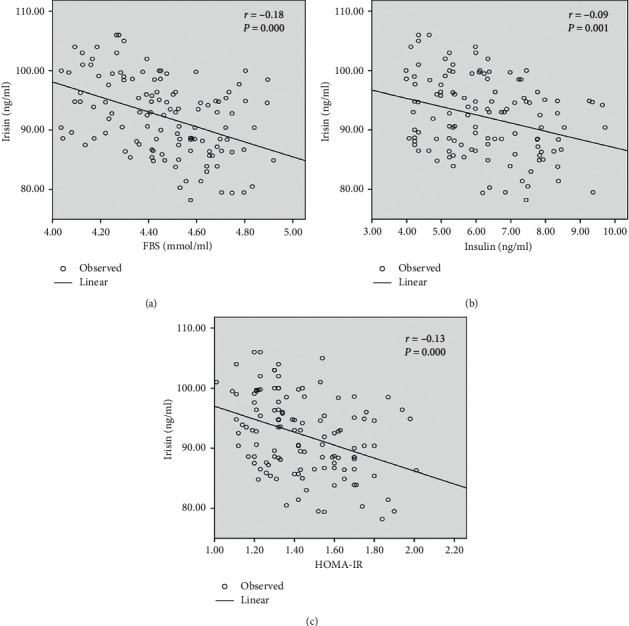
Correlation between irisin and metabolic variables after six months of moderate physical exercise in the normal weight group.

A correlation between irisin levels and other metabolic parameters after six months of physical exercise in normal weight and obese groups was studied by the Pearson correlation. As it is shown in Table 3 and Figures 1 and 2, a significant negative correlation was demonstrated between the irisin level and FBS and insulin and HOMA-IR in the normal weight group. Interestingly, there were negative significant differences between irisin and all metabolic variables, BMI, WC, FBS, insulin, HOMA-IR, and HOMA-B_2_, in the obese group.

## 4. Discussion

It is well-known that physical exercise has a significant role in treating obesity. Physical exercise induces browning of white adipose tissue and stimulates metabolic gene expressions that have a role in oxidative phosphorylation and increased energy expenditure [7, 16, 17]. Irisin is a secreted hormone that cleavages from FNDC 5 protein which is encoded by an exercise-induce gene, Fndc1 [7, 18]. Even though irisin is known as exercise-induced myokine, some researches reveal different results. These conflicting researches may be because of differences between the heaviness of loading and duration of physical exercise and age of participants [19, 20]. Thus, the main goal of this study is to investigate the effect of six months of physical exercises on irisin in normal weight and obese subjects.

The results in the present study revealed that there are high significant differences in each BMI, WC, FBS, insulin, HOMA-IR, and HOMA-B_2_ between the normal weight group and obese group, suggesting that obesity has a role in impair of glucose-insulin homeostasis. Consistent with the previous study [8, 12], there is a significant increase in the irisin level in the obese group compared to the normal weight group. The potential explanation could be because hypersecretion of irisin in the obese group is as a compensatory mechanism to combat irisin resistance in the obese [12, 16, 17, 21]. Also, other studies had revealed that irisin is secreted either by an regulatory mechanism between muscle and adipose tissue or by a positive feedback mechanism from adiposity and through itself by an autocrine effect [22, 23]. In addition, there is a significant increase in the irisin level before and after physical exercise in both normal weight and obese group. This result is in agreement with the work of Bostrom et al. and other studies which support the cross talking between muscle contraction and adipose thermogenesis [7, 23–25].

It is well established that obesity has a significant role in disturbing the metabolic regulation of glucose which is characterized by elevated insulin levels, glucose tolerance, and HOMA-IR [26]. This study demonstrated that six months of moderate physical exercise was related to improved glucose tolerance which was clearly interpreted by a significant decrease in fasting blood glucose, insulin, and HOMA-IR before exercise compared to after exercise in both normal weight and obese participants. Decreased insulin levels were not concurrent with increased glucose levels indicating a potential improvement of glucose metabolism and glucose uptake. The results of this study are supported by previous studies which suggested that moderate intensities of physical exercise produce beneficial improvements in insulin sensitivity and glucose tolerance in obese subjects [23, 27]. To support this finding, significant negative correlations were shown between exercise-induce irisin levels and BMI, fasting blood glucose, insulin, HOMA-IR and HOMA-B_2_ levels in obese group, suggesting that increase of irisin levels had significantly improved, fasting blood glucose, fasting insulin, insulin resistance, and beta-cell function after six months of physical exercise. In contrary to the present study, it was shown that there is a positive correlation between irisin levels and BMI in some studies [12, 19], irisin, and HOMA-IR [28]. This disagreement could be because of the different number of individuals in different studies or the difference in the length of the physical exercise program.

This study demonstrated that there are no significant differences in the BMI and WC between before and after six months of exercise in the normal weight group. Moreover, the irisin level does not significantly correlate with an adiposity biomarker; BMI, and WC, whereas irisin has a significantly negative correlation with insulin, FBS, and HOMA-IR and slightly, but not significantly, correlated with HOMA-B_2_. This means that the improvement in insulin sensitivity and glucose tolerance is due to the increase in irisin levels, not due to weight reduction. Further studies are needed to investigate the mechanism between irisin and glucose/insulin metabolism in nonobese subjects after a long term of physical exercise.

The limitations of the present study include the following: firstly, it lacks the evidence of browning markers. Secondly, it does not include body composition parameters such as, fat mass, body muscles, and fat-free mass.

## 5. Conclusions

Together, this study concluded that a long term of moderate physical exercise significantly increases circulating irisin levels in both obese and normal weight subjects. In addition, irisin was negatively correlated with fasting blood glucose, insulin, insulin resistance, *β*-cell function and adiposity markers, BMI, and WC, suggesting that elevated irisin could have beneficial effects on regulation glucose, insulin resistance, and obesity. Thus, further investigations are needed to assess the role and mechanism of physical exercise-induced irisin on body composition including muscle mass and fat mass and to assess the utility of irisin as a treatment in obesity.

## Figures and Tables

**Table 1:** Differences of metabolic variables and irisin in normal weight and obese groups in each four assessments and during six months of physical exercises.

Variables	Months	Normal weight	Obese	*P* value
*BMI (kg/m* ^*2*^)				
	0	23.00 ± 0.62	42.98 ± 1.80	**0.0001**
	2	22.86 ± 0.68	42.56 ± 1.60	**0.0001**
	4	22.77 ± 0.70	41.38 ± 1.43	**0.0001**
	6	22.65 ± 0.88	35.85 ± 1.66	**0.0001**
*P* value		NS	NS	

*WC (cm)*				
	0	76.31 ± 0.80	104.16 ± 3.98	**0.0001**
	2	76.11 ± 0.78	103.89 ± 3.30	**0.0001**
	4	75.97 ± 0.75	103.34 ± 1.75	**0.0001**
	6	75.94 ± 0.78	100.11 ± 3.09	**0.0001**
*P* value		NS	NS	

*FBS (mmol)*				
	0	4.71 ± 0.10	6.98 ± 0.19	**0.0001**
	2	4.54 ± 0.11	6.65 ± 0.14	**0.0001**
	4	4.42 ± 0.08	6.63 ± 0.16	**0.0001**
	6	4.17 ± 0.08	6.11 ± 0.08	**0.0001**
*P* value		**0.005**	NS	

*Insulin (ng/ml)*				
	0	7.83 ± 0.87	24.48 ± 2.72	**0.0001**
	2	6.15 ± 1.49	22.07 ± 1.78	**0.0001**
	4	5.84 ± 0.91	20.51 ± 1.96	**0.0001**
	6	5.04 ± 0.69	16.14 ± 2.81	**0.0001**
*P* value		NS	**0.05**	

*HOMA-IR*				
	0	1.60 ± 0.20	7.57 ± 1.18	**0.0001**
	2	1.51 ± 0.20	6.65 ± 1.11	**0.0001**
	4	1.36 ± 0.16	5.29 ± 0.69	**0.0001**
	6	1.26 ± 0.14	4.13 ± 0.51	**0.0001**
*P* value		**0.05**	**0.005**	

*HOMA-B*_*2*_				
	0	129.98 ± 17.47	140.92 ± 17.59	**0.0001**
	2	119.44 ± 33.28	140.31 ± 13.35	**0.0001**
	4	127.54 ± 21.78	131.48 ± 15.33	**0.0001**
	6	152.19 ± 30.66	123.27 ± 20.59	**0.0001**
*P* value		NS	**0.05**	

*Irisin (ng/ml)*				
	0	87.54 ± 6.08	91.10 ± 4.71	**0.05**
	2	90.56 ± 4.79	107.01 ± 6.40	**0.0001**
	4	93.46 ± 5.17	124.32 ± 6.68	**0.0001**
	6	97.39 ± 5.68	139.50 ± 6.96	**0.0001**
*P* value		**0.01**	**0.0001**	

BMI: body mass index, WC: waist circumference, FBS: fasting blood sugar; HOMA-IR: homeostatic model assessment of insulin resistance index, HOMA-B2: homeostatic model assessment of insulin of pancreatic cells-beta, NS: nonsignificant.

**Table 2:** Differences of irisin and metabolic variables before and after six months of physical exercise.

Variables		Before exercise mean ± SD	After exercise mean ± SD	*P* value
BMI (kg/m^2^)	Normal weight	23.00 ± 0.62	22.65 ± 0.88	0.091
	Obese	42.98 ± 1.80	35.85 ± 1.66	**0.0001**

WC (cm)	Normal weight	76.31 ± 0.80	75.94 ± 0.78	0.103
	Obese	104.16 ± 3.98	100.11 ± 3.09	**0.0001**

FBS (mmol/l)	Normal weight	4.71 ± 0.10	4.17 ± 0.08	**0.0001**
	Obese	6.98 ± 0.19	6.11 ± 0.08	**0.0001**

Insulin (ng/ml)	Normal weight	7.83 ± 0.87	5.04 ± 0.69	**0.0001**
	Obese	24.48 ± 2.72	16.14 ± 2.81	**0.0001**

HOMA-IR	Normal weight	1.60 ± 0.20	1.26 ± 0.14	**0.0001**
	Obese	7.57 ± 1.18	4.13 ± 0.51	**0.0001**

HOMA-B_2_	Normal weight	129.98 ± 17.47	152.19 ± 30.66	**0.001**
	Obese	140.92 ± 17.59	123.27 ± 20.59	**0.001**

Irisin (ng/ml)	Normal weight	87.54 ± 6.08	97.39 ± 5.68	**0.0001**
	Obese	91.10 ± 4.71	139.50 ± 6.96	**0.0001**

**Table 3:** Correlation between irisin and other metabolic variables in normal weight and obese groups.

Variables	Normal weight group		Obese group	
	*r*	*P* value	*r*	*P* value
BMI (kg/m^2^)	−0.20	0.25	−0.50	**0.0001**
WC (cm)	−0.007	0.36	12-0.	**0.0001**
FBS (mmol/l)	−0.18	**0.0001**	0.61-	**0.0001**
Insulin (ng/ml)	−0.09	**0.001**	0.56-	0.0001
HOMA-IR	− 0.13	**0.0001**	−0.59	**0.0001**
HOMA-B_2_	0.02	0.064	−0.16	**0.0001**

## Data Availability

No data were used to support this study.
